# Hepatitis B Vaccination in Chronic Kidney Disease: Review of Evidence in Non-Dialyzed Patients

**DOI:** 10.5812/hepatmon.7359

**Published:** 2012-11-14

**Authors:** Alicja E. Grzegorzewska

**Affiliations:** 1 Department of Nephrology, Transplantology and Internal Diseases, Poznań University of Medical Sciences, Poznań, Poland

**Keywords:** Vaccination, Hepatitis B Virus, Kidney Disease

## Abstract

**Context:**

Hepatitis B vaccination of hemodialysis patients is performed all over the world. There are also recommendations from world health organizations to vaccinate patients with chronic kidney disease (CKD) prior dialysis commencement, but the implementation of a hepatitis B vaccination program is less common and not well organized.

**Evidence Acquisition:**

This review article summarizes data indicating why, when and how to vaccinate CKD patients before they start renal replacement therapy. Publication for this review was bringing into being from PubMed.

**Results:**

There is an agreement in the nephrological societies and among clinicians and scientists that CKD patients should be vaccinated in early stages of their disease, because a higher glomerular filtration rate is more likely to be associated with the responsiveness to vaccination. Schedules of vaccination and optimal vaccine doses are still being investigated. Differences in data with respect to these problems may result from comparisons of various vaccine doses and vaccination schedules without reference to one gold standard, variations in patients` clinical status and glomerular filtration rate, and also the small groups of the affected patients make statistical analysis non-conclusive. A titer of antibodies to surface antigen of hepatitis B virus (anti-HBs) > 10 IU/L or ≥ 10 IU/L is commonly considered as a marker of seroconversion to anti-HBs positivity after vaccination in both non-dialyzed and dialyzed patients. In advanced CKD, vaccine–induced serconversion rate is seldom observed in more than 90% of vaccinees. Various strategies have been utilized in order to increase vaccine-induced seroconversion rate in patients with advanced CKD. Changing the injection mode, the use of adjuvants and immunostimulants to improve the immunogenicity of existing recombinant hepatitis B vaccines, introduction of mammalian-cell derived pre-S/S HBV vaccines (third-generation vaccines) were tried in order to improve the immunization rate.

**Conclusions:**

There are a substantial number of non-responders to the hepatitis B vaccine among CKD patients. Therefore, successful prevention of hepatitis B virus transmission and spread will only be attained when hepatitis B vaccination is applied together with full implementation of appropriate infection control procedures.

## 1. Context

Prevention of infectious diseases is an important problem worldwide. Hepatitis B may be largely preventable by vaccination. Administration of hepatitis B virus (HBV) surface antigen (HBsAg) in recombinant vaccines leads to the development of protective antibodies to HBV (anti-HBs) in responders. Lack of the development of anti-HBs means vaccinees is susceptible to HBV infection. Special population groups, among them patients with chronic kidney disease (CKD) requiring renal replacement therapy (RRT), essentially hemodialysis (HD), are already recognized as a risk group. Strict procedures controlling transmission of blood-born infections during HD sessions as well as hepatitis B vaccination are recommended and are introduced on mandatory basis in most HD centers worldwide. The Center for Disease Control and Prevention in Atlanta (USA) has recommended hepatitis B vaccination of HD patients since 1982 (1). In the United Kingdom (UK), vaccination of HD patients likely to have dialysis abroad was also recommended in 1982 by the Advisory Group in Hepatitis (2). However, in the UK in 1987-1991, most (63.8%) of the analyzed dialysis units routinely immunized staff but only 5% routinely immunized patients (3). Among HD patients susceptible to HBV, the two-year risk of seroconversion for HBV infection was 38.9%, accounting for 19 seroconversions to HBsAg positivity per 100 patient-years (4). In 1999 it was reported from the USA that the risk for HBV infection was 70% lower in vaccinated HD patients compared to those who did not receive the vaccine (5). During 1997–2002, the percentage of patients vaccinated against HBV infection in the USA increased from 47% to 56% (6). The prevalence of HBV infection in USA HD patients, defined as the percentage of all HD patients who tested positive for HBsAg, progressively fell from 7.8% to 1.0% between 1976 and 2002 (6). In the last two decades, the incidence of HBV infection during HD treatment, defined as the percentage of all patients receiving HD during the data collection period who seroconverted from HBsAg negative to HBsAg positive, decreased remarkably worldwide mainly due to implementation of hepatitis B vaccination in the majority of HD patients. Recent data indicate that HBsAg positivity de novo accounts for about 0.15 episodes per 100 patient-years (7-9). However, the true infection rate should also include incidence of positivity to antibodies to HBV core antigen (anti-HBc). Such an incidence was evaluated as 2.35 episodes/100 patient-years (7). Results of hepatitis B vaccination in patients already requiring RRT are, however, not fully satisfactory, because patients with advanced CKD typically show an impaired immune response to hepatitis B vaccination compared to healthy individuals. Immune and non-immune low responsiveness to viral antigens shown in HD patients as compared with individuals without impaired renal function results in the former group in a lower frequency of anti-HBs development. Compared to a response rate of over 90% in the general population (10), only 50 to 85% of dialysis patients achieve antibody levels conferring protection [> 10 IU/L (11)] following hepatitis B vaccination (10, 12-18). Lower responsiveness to hepatitis B vaccination occurs despite recommendations to use higher vaccine doses in HD patients than in general population (19). Moreover, an anti- HBs titer tends to fall with time in persons who mounted an antibody response. In dialysis patients, the loss of hepatitis B immunity seems to be quicker than in healthy subjects (10, 16, 18). Ineffective vaccination is predictive for prevalence and incidence of HBsAg positivity (4) and anti-HBc positivity (7, 20). At present, the attention of nephrologists is focused on CKD patients, who are currently non-dialyzed, but as their kidney disease progresses, it is likely to lead to RRT in the future. Hepatitis B vaccination of such patients is thought to decrease a number of HBV susceptible patients on RRT. The purpose of this overview is to summarize data on hepatitis B vaccination in CKD patients not requiring RRT (dialysis or renal transplantation) and to show arguments answering the question as to why we should to vaccinate patients in early CKD stages.

## 2. Evidence Acquisition

Publications for this review, not the time duration of search limited, were found in PubMed in response to catchwords: “hepatitis B vaccination, pre-dialysis” (5 citations), “hepatitis B vaccination, chronic kidney disease” (196 citations), “hepatitis B vaccination, dialysis” (372 citations), and “hepatitis B vaccination, kidney transplantation” (76 citations). Articles, selected for citation in this review, were chosen on the basis of their originality and efficacy in discussion on advantages/disadvantages of hepatitis B vaccination in early CKD stages. Results of the literature review were organized in various parts concerning facts on factors involved in non-responsiveness to HBV vaccination (1), results of hepatitis B vaccination in different CKD stages (2), prevention of HBV infection prior dialysis commencement (3), anti-HBs decline and a response to booster doses (4), methods of hepatitis B vaccination in non-dialyzed CKD patients (5), protective anti-HBs titer (6) trials for improvement of vaccine-induced seroconversion rate (7), future options (9), and summary and conclusions (8).

## 3. Results

### 3.1. Factors Involved in Non-responsiveness to HBV Vaccination

Numerous inherited and/or acquired factors are implicated in diminished immunization following hepatitis B vaccination. However, at first we should exclude variables such as improper storage or administration that is not compatible with a manufacturer instruction. Involvement of genetic factors in the anti-HBs development is continuously examined. Already in the 70-ties of the past century, immune response to HBsAg in HBV infected HD patients was linked to human leukocyte antigens (HLA) (21, 22). In 1985 Hashimoto et al. (23) linked HLA with anti-HBs response to hepatitis B vaccine. In 1988 HLA-linked immune suppression gene controlling the non-responsiveness to HBsAg through HBsAg-specific suppressor T cells was found in Japanese population (24). In Caucasians, an association between the HLA-B8, DR3 haplotype and low responsiveness to HBsAg was noted (25), whereas an association between the HLA-DR1 and high responsiveness to HBsAg was described in both Caucasian (26) and Japanese persons (27). In 1990, HD patients, identified as non-responders, had shown to have a higher frequency of HLA-A1, B8 and DR3 than non-responders (15). In 1998 Hohler and et al. (28) have reported enhanced expression of DRB 1×3, DRB 1×7 and DRB 1×14 in nonresponders to hepatitis B vaccine. More recently, interleukin genotypes (examples IL10, IL-12, IL-18) were associated with the anti-HBs development in response to HBsAg in HD patients (29-31). A very well established negative factor of immunization failure is increasing age (30, 32-34). Seroconversion rate to anti-HBs positivity after vaccination was 84% in HD patients below 40 years and only 33% in those ≥ 60 years (34). The impaired response to hepatitis B vaccine in dialysis patients has been also attributed to male gender (15, 35), poor nutritional status, mainly low serum albumin concentration (36, 37), serological positivity for hepatitis C virus (HCV) (38) or human immunodeficiency virus (HIV) (39), and diabetes mellitus (29, 40). Vitamin D deficiency was associated with a poor antibody formation upon hepatitis B vaccination in stage 3-5D CKD patients (41). It is well known that dialysis patients are immunocompromised. Severity of immune abnormalities increases with deterioration of renal function. The best example is correlation of serum soluble CD40 levels with creatinine in non-dialyzed CKD patients. Soluble CD40 is able to inhibit immunoglobulin production by CD154-activated B lymphocytes in vitro. HD patients presented a fivefold increase in soluble CD40 compared to healthy subjects, whereas non-dialyzed CKD patients showed a three-fold increase. Furthermore, the positive correlation was observed between the serum levels of soluble CD40 and the deficient response to hepatitis B vaccination (42). These and other data showing deterioration of the immune system in the course of CKD should be a strong reason for vaccination of CKD patients in initial stages of their renal diseases. Increasing prevalence of immune abnormalities and co-morbidities with severity of CKD, which decreases vaccine-induced immunization, leads to the conclusion that hepatitis B vaccination before dialysis commencement may benefit in the better protection against HBV when patients start RRT.

### 3.2. Results of Hepatitis B Vaccination in Different CKD Stages

In 1988, Seaworth et al. (43) published that seroconversion rates in excess of 80% can be achieved if patients are given a vaccine prior the onset of dialysis dependence. When they analyzed results of currently available evidence, it was shown that patients with renal failure not yet dependent on dialysis respond more favorably to the plasma-derived vaccine than dialyzed patients (80 vs 50%). In 1999, Agarwal et al. (44) demonstrated very convincingly that CKD patients should be vaccinated at the very early stage of CKD. They showed that seroconversion rate among patients with mild (creatinine 1.5 to 3.0 mg %), moderate (creatinine 3.0 to 6.0 mg %) or severe (creatinine > 6.0 mg %) CKD was 87.5%, 66.6%, and 35.7% after three 40 µg vaccine doses, respectively, and 100%, 77%, and 36.4%, respectively, following four 40 µg vaccine doses. In 2003, DaRosa et al. (45) have shown that CKD stage predicts seroconversion after hepatitis B immunization and also concluded that to vaccinate earlier would be beneficial. Authors prospectively analyzed 165 patients with median estimated glomerular filtration rate (GFR) of 20 (interquartile range, 14 to 20) mL/min. Seroconversion rate was 82%. Level of GFR showed the independent positive predictive value for seroconversion in multivariate analyses.

In the study by Hashemi et al. (14) a higher estimated GFR was not associated with significantly improved seroconversion, but their data (not adjusted for significant differences in age, body mass, and hemoglobin between groups) also indicate statistically borderline decrease (P = 0.067) in positive response with deteriorating GFR: 80% in CKD stage 3, 76% in CKD stage 4 and 67% in CKD stage 5. Moreover, data by Hashemi et al. (14) showed a significant positive correlation between an anti-HBs titer and estimated GFR. CKD stage, however, was not associated with seroconversion in the multivariate analysis. When seroconversion rate in CKD stage 5 is compared to that attained in CKD stage 5D, differences are usually not-significant. Bel’eed et al. (46) demonstrated that the vaccine-induced seroconversion rates were similar in predialysis patients with serum creatinine > 400 µmol/L (68%), HD patients (66%), and peritoneal dialysis patients (66%). For comparison, serological response to hepatitis B vaccine in HIV-infected children was 59.5% (47). Differences in aforementioned data may result from various vaccine doses and vaccination schedules, variations in patients’ clinical status and GFR, and likewise from small groups of the examined patients, makes statistical analysis non-conclusive. Experts’ opinions, which based on currently available evidence and experience, resulted in recommendations of hepatitis B vaccination before dialysis commencement. In 1988 in the UK it was recommended that patients with chronic renal failure should receive hepatitis B vaccine early in the course of their disease (48). Guidance from the UK Health Departments in 1991 suggested that CKD patients should “be immunized as soon as it becomes likely that they will ultimately require treatment by maintenance haemodialysis or renal transplant” (49). According to European Best Practice Guidelines (2002) “patients with progressive renal failure should be vaccinated against HBV preferably before the start on HD” (11). Although immunization rates not always seem to be much higher and stronger in non-dialysis patients [67 – 100% (14, 43-46, 50, 51)] than in those already dialyzed [50 – 85% (10, 12-18, 46)], hepatitis B vaccination of CKD patients should be advocated in early stages of the kidney disease for other several reasons shown in the next parts of this review. 

### 3.3. Prevention of HBV Infection prior Dialysis Commencement

CKD patients entering dialysis programs frequently demonstrate serological markers of HBV transmission. In our study HBV infection in 82% of HBsAg positive patients was not associated with HD treatment, for the reason that it was acquired before HD commencement (13). The total rate of HBV seromarkers was 20.9% in patients starting HD therapy in Lima hospital. HBsAg positive patients amounted for 2.3% of all new HD persons; such patients usually replicate HBV and may be a potential source of infection for other HD patients. Anti-HBc positivity with negative HBsAg (or decreased to an undetectable level) reached 18.7% in patients using the HD program for the first time (52). Although such patients are routinely considered as having HBV transmission in the past and not infectious to others, there is an increasing evidence that these persons may replicate HBV (occult HBV infection) or may start to replicate under special circumstances (immunosuppression, cachexia). Vaccination in early CKD stages may help to avoid HBV infection before the start of dialysis and to decrease a risk of HBV transmission in dialysis centers, where such a possibility is much higher than in noninvasive healthcare facilities.

### 3.4. Anti-HBs Decline and a Response to Booster Doses

A time-related anti-HBs decline is well known phenomenon. In the general population, hepatitis B vaccination provided strong protection against infection for at least 15 years in all age groups, although antibody levels decreased the most among individuals immunized at 4 years of age or younger (53). In children, loss of anti-HBs was observed in 18.4% after 5 years (54). In the general population, a greater anti-HBs decline in adult females than males was shown in the study by McMahon et al. (53). In dialyzed patients, loss of anti-HBs is more rapid than in the general population. A year since standard vaccination, (3 × 20 µg) of healthy persons 77.2 – 82.5% of vaccine recipients had an anti-HBs titer > 10 IU/L, whereas HD patients having 4 × 40 µg vaccination schedule showed the positive titer in 53.3% of cases (10). In another study, 26% of HD patients lost immunity during 6 – 36 months of observation (18). The waning of protective anti-HBs antibodies was detected in 47% and 68% of dialysis patients during 3 and 5 years following vaccination, respectively (16). A quicker decrease or loss of anti-HBs in dialysis patients compared to the healthy population may be related to the reduced anti-HBs levels immediately after vaccination and impaired immune status in the former group. Healthy immunocompetent individuals usually do not need monitoring of anti-HBs and booster doses when they were effectively immunized by the primary hepatitis B vaccination series (55), especially when their documented anti-HBs titer was > 100 IU/L. In vaccinated HD patients, anti-HBs should be followed-up regularly to give a boosting dose of HBV vaccine whenever anti-HBs titer approaches 10 IU/L. According to the European Best Practice Guidelines for HD patients, anti-HBs should be checked every 6 – 12 months (11). American Association for the Study of Liver Diseases recommends annual follow-up testing of hemodialyzed vaccine responders (56). Efficacy of a response to booster doses may be of importance for making decisions when to vaccinate CKD patients. Dukes et al. (57) have observed that progression to dialysis was associated with poorer initial response to vaccination compared with the response in patients remaining dialysis-independent, but the response to booster immunization was favorable in both groups. They concluded that immunization of predialysis patients and subsequent booster vaccine resulted in a more favorable antibody response than has been seen in HD patients. Pre-emptive renal transplantation has become increasingly popular. Candidates for such a method of RRT should be vaccinated against HBV in the pre-transplant period. Renal allograft recipients on immune suppressants, even vaccinated with an enhanced scheme (4 × 40 µg of the recombinant vaccine), show low seroconversion rate [36% in the study by Lefebure et al. (58)]. Patients with poor renal graft function (serum creatinine > 400 µ mol /L) had lower seroconversion rate than CKD patients with serum creatinine > 400 µmol/L (41% vs. 68%) (46). On the other hand, recipients vaccinated before transplantation developed anti-HBs in 86% of cases in response to a booster injection (58).

### 3.5. Methods of Hepatitis B Vaccination in Non-dialyzed CKD Patients

There are detailed recommendations how to vaccinate general population and dialysis patients. Standard hepatitis B vaccination schedule, commonly used in the general adult population means three 20 µg licensed conventional recombinant vaccine doses at zero, one and six months. HD patients should be vaccinated against HBV using licensed conventional recombinant HBV vaccines given at zero, one, two and six months in the dose of 40 µg each. A vaccine has to be administered by the intramuscular route at one site. Patients who did not respond to the primary vaccine series should be revaccinated with three additional doses and retested for response (1). Four doses of the recombinant vaccine (40 µg each) were given to HD patients prior establishment of these recommendations and are at present commonly used worldwide (10, 18). The deltoid muscle is the preferred injection site; gluteal injections have been associated with lower response rates, because a vaccine was probably injected to fat rather than muscle (59, 60). Various immunization strategies have been developed for hepatitis B vaccination of non-dialyzed CKD patients, but none are commonly accepted. Available data reveals that in non-dialysis CKD patients there is a need for trials evaluating modification of standard hepatitis B vaccination (3 × 20 μg at zero, one and six months) towards increased doses, comparable to those used in dialysis patients. Agarwal et al. (44) compared two hepatitis B vaccination schedules in patients with mild (creatinine 1.5 to 3.0 mg%), moderate (creatinine 3.0 to 6.0 mg%), and severe (creatinine > 6.0 mg%) CKD. Doses of 40 μg of recombinant vaccine Engerix (20 μg in each deltoid region) were given at zero, one and two months (3-dose group) or at zero, one, two, and six months (4-dose group). In the 3-dose group, the seroconversion rate among patients with mild, moderate, and severe CKD was 87.5%, 66.6%, and 35.7%, respectively, whereas in the 4-dose group it was 100%, 77%, and 36.4%, respectively. They concluded that patients with CKD should be vaccinated using 40 μg of vaccine, and that 4 doses is better than 3 doses (44). In the study by McNulty et al. (61), Engerix B recombinant vaccine doses of 40 μg administered at 0, 1 and 6 months to predialysis patients with moderate chronic renal failure attained equivalent seroconversion to 3 doses of 20 μg (67% vs. 57%, p = 0.27). However, this 10% difference is significant, if the same seroconversion rate is maintained in 5 times greater groups (P = 0.015, the Fisher test). Ahmadi et al. (50) have found that in CKD patients 3 doses of 20 μg did not indicate a significantly different seroconversion rate compared to 4 doses of 40 μg (92% vs. 80.9%, P = 0.41). On the other hand, in the same study, they have shown that the 4-dose hepatitis B vaccine (40 μg each) resulted in the significantly greater seroconversion than 3 doses of 40 μg (80.9% vs. 77%, P = 0.004). This inconsistency may result from the small number of studied patients. In summary, a majority of the aforementioned results indicates directly or indirectly that higher doses of vaccine generate higher seroconversion rates.

### 3.6. Protective Anti-HBs Titer

Vaccinees that developed an anti-HBs titer above two and below 10 IU/L are sometimes referred to as hyporesponders. An anti-HBs titer of > 10 IU/L or ≥ 10 IU/L is commonly considered as a marker of positive seroconversion, but vaccinees that developed an anti-HBs titer between 10 and 100 IU/L are sometimes referred to as low responders. In the UK, the optimum response, conferring seroprotection against HBV infection, was defined at an anti-HBs titer ≥ 100 IU/L (62). An anti-HBs titer > 10 IU/L is also referred as a “protective” titer, although it may not confer protection after HBV transmission in every case. Renal transplant recipients underwent HBV infection from HBsAg positive renal allografts despite having anti-HBs acquired by vaccination (63). Occurrence of an anti-HBs titer > 10 IU/L in 67% of patients who seroconverted to anti-HBc positivity also suggests that such an anti-HBs titer does not always protect against HBV infection in HD patients (7). Lombardi et al. (64) postulated that in dialysis patients an anti- HBs titer > 50 IU/L should be considered as protective. In some groups of patients, like HBsAg negative children awaiting liver transplantation, an anti-HBs titer > 200 IU/L was advised to be sufficient to prevent de novo HBV infection (65). Fabrizi et al. (66) have listed a few circumstances under which a level of 10 IU/L might not completely exclude HBV infection. These would include exposure to an overwhelming HBV dose (67), production of antibody recognizing an HBsAg determinant different from that common to all subtypes (68, 69), or infection by an HBV mutant producing HBsAg with determinants not neutralized by anti-HBs (70, 71).

### 3.7. Trials for Improvement of Vaccine-induced Seroconversion Rate

Various strategies have been utilized in the past to increase the vaccine-induced seroconversion rate in patients with advanced CKD. Changing the injection mode (the intradermal route vs. the intramuscular route or the combined use of the intradermal and intramuscular routes) was tried, also in predialysis patients (72-74), showing controversial results. Hepatitis B vaccination scheme involving the combined use of the intradermal and intramuscular routes, elaborated by Marangi et al. (73) in non-dialyzed and dialyzed patients with serum creatinine concentration ≥ 4 mg/dL, yielded very promising effects (all patients developed anti-HBs); however, it did not become widely popular. In 1984, Milich et al. (75) showed that enhanced immunity to HBsAg can be induced using pre-S antigens in non-responder mice resistant to protein S of HBsAg. Third-generation vaccines, which contain mammalian-cell derived pre - S1 / pre - S2 / S have been developed. Pre-S antigens seem to induce neutralizing antibodies, which block attachment, endocytosis and possibly membrane penetration of HBV into the hepatocyte (76). Pre-S/S vaccines should provide faster and more augmented seroconversions rates compared to recombinant vaccines. The immunogenicity of third-generation vaccine was demonstrated in 120 pre-dialysis patients who received 5-doses of either 20 µg/dose of the pre - S2 / S Gen Hevac B® vaccine or 5 µg / dose of the Pasteur plasma-derived vaccine at zero, one, two, four and 12 months (51). The recombinant vaccine elicited the higher seroconversion rate (anti-HBs titer ≥ 2 IU/L) as compared to the plasma-derived vaccine (94 vs. 76%), but difference in seroconversion to anti- HBs titer ≥ 10 IU/L was borderline (84 vs. 70%, p = 0.053). The use of adjuvants to recover the immunogenicity of existing recombinant HBV vaccines is suggested from results of experimental studies. Hepatitis B recombinant DNA vaccine adjuvanted by AS04C containing 3-0-desacyl-4’-monophosphoryl lipid A adsorbed on aluminium phosphate (Fendrix) was reported be more effective in HD patients than the older recombinant vaccine (Engerix) (77). Three doses of the investigational AS02 (v)-adjuvanted hepatitis B vaccine HB-AS02 have been shown to induce still more rapid seroprotection and higher anti-HBs antibody concentrations in CKD patients than four doses of Fendrix (78). This vaccine was also reactogenic in 76.9% of CKD patients who failed to respond to prior vaccination with a conventional hepatitis B vaccine and provided higher anti-HBs concentrations following a booster dose than a conventional vaccine (79). However, Fabrizi et al. (80) showed in their meta-analysis of prospective randomized trials that the immune response to recombinant hepatitis B vaccine continues to be unsatisfactory despite adjuvantation. Co-administering levamisole p.o. (17), the use of polymethylmethacrylate dialyzers able to remove serum high molecular weight toxins, among them soluble CD40 (81), supplementation with zinc aspartate after each dialysis session (82), the use of thymopentin s.c. (83), granulocyte macrophage colony stimulating factor (84), IL-2 (85) were also tried to strengthen the antibody response. Treatment with recombinant human erythropoietin was associated with increased antibody titers after hepatitis B vaccination in dialysis patients (86). Treatment with a vitamin D receptor activator had no influence on the anti-HBs development (41).

### 3.8. Future Directions

Genetic investigation could help in the development of improved hepatitis B vaccines and may eventually reduce the proportion of vaccine failures (87). Positive response to vaccines was shown to be associated with increased interferon (IFN)-gamma production (88, 89). As IL-18 is involved in IFN-gamma production (90-92), it was used to modulate DNA vaccines against HBV (89, 93, 94). Channarong et al. (93) have constructed a recombinant plasmid carrying gene encoding HBsAg linked to DNA segment encoding full-length murine IL-18. All vaccinated mice revealed a significant serum anti-HBsAg IgG response after two intramuscular injections of the vaccines as compared to the level of mice vaccinated without DNA segment encoding IL-18. It is conceivable that in the near future all people worldwide will be vaccinated on the mandatory basis. The World Health Organization has recommended that hepatitis B vaccination should be included in a worldwide routine immunization for all children. Cost-benefit analyses have strongly supported the introduction of universal vaccination against HBV to newborns (95, 96). Results of children’s vaccination, which were evaluated in the six-year outcome of the program, showed neither new cases of HBsAg de novo nor seroconversion to anti-HBc positivity (54). 

## 4. Conclusions

There are several reasons for hepatitis B vaccination of CKD patients in early stages of their chronic disease:

1) Less severe immune abnormalities in early CKD stages give a chance for higher seroconversion rates,

2) Immunization of RRT dependent patients is less satisfactory than that shown in non-dialyzed CKD patients,

3) An immune response to vaccination is stronger in early CKD stages,

4) If patients are vaccinated early, pre-dialysis HBV infection may be avoided,

5) A response to booster doses in patients responding for the primary vaccination in early CKD stages is usually maintained during RRT.

Results of hepatitis B vaccination in non-dialyzed CKD patients are summarized in Table 1. The best results of vaccination are shown up to CKD stage 3 - 4. In CKD stage 5 results are often not significantly different from those attained in CKD stage 5D. A vaccination schedule is not established for non-dialyzed CKD patients, but more advanced schedules should be taken into account, especially in later CKD stages. A full success in prevention of HBV transmission and spread is obtained when an implementation of prophylactic education and appropriate infection control procedures are applied together with vaccination.

**Table 1:** Main Results of Hepatitis B Vaccination in Non-Dialyzed CKD Patients

	Description of Renal Function	SR, %	Main Results
**1988 (43)**	PCR: mean 4.5 (range 2.0 – 9.8) mg/dL	42-81	A higher SR for plasma-derived than for recombinant vaccine
**1994 (51)**	GFR: 25.3 ± 12.6 mL/min for pre-S2/S vaccine and GFR: 24.4 ± 11.1 mL/min for plasma-derived vaccine	76-94	A higher SR with anti-HBs titer ≥ 2 IU/L was for pre-S2/S vaccine than for plasma-derived vaccine
**1999 (44)**	PCR: 1.5 – 3.0 mg/dL Pcr: 3.0 – 6.0 mg/dL PCR: > 6.0 mg/dL	87.5-100 66.6-77 35.7-36.6	SR was negatively dependent on PCR and positively on vaccine dose
**2002 (46)**	PCR: > 400 µmol/L	68	SR was similar in pre-dialysis, HD and PD patients
**2003 (45)**	GFR: median 20 (interquartile range 14 to 20) mL/min	82	The higher GFR the higher SR
**2003 (47)**	PCR: 5.6 ± 0.4 mg/dL	85.7-89.5	ID vaccination at a lower dose provided comparable SR like IM route at double the standard dose
**2005 (61)**	PCR: 200 - 600 µmol/L	57-81	SR was dependent neither on GFR nor vaccine dose
**2011 (14)**	GFR: 27.7 ± 14.7 mL/min/1.73 m ^2^	78	SR was not dependent on GFR, but anti-HBs titer positively correlated with GFR
**2012 (41)**	CKD stage 3 – 5D GFR: 22.8 ± 8.5 mL/min/1.73 m ^2^ in non-dialysis patients	57 (in the entire group)	SR and anti-HBs titer were positively dependent on vitamin D levels Dialysis/pre-dialysis status was not a predictor of SR
**2012 (50)**	CKD stage 3 - 4	77-92	No differences in SR dependent on vaccination schedule (4 × 40 µg vs. 3 × 20 µg)

Abbreviations: anti-HBs: antibodies to surface antigen of hepatitis B virus, CKD: chronic kidney disease, GFR: glomerular filtration rate, HD: hemodialysis, ID: intradermal, IM: intramuscular, Pcr: plasma creatinine concentration, PD: peritoneal dialysis, SR: seroconversion rate.
